# Association of the Uric Acid-to-Magnesium Ratio and Frontal QRS-T Angle with Angiographic Coronary Disease Severity in Acute Coronary Syndrome

**DOI:** 10.3390/jcm15176920

**Published:** 2026-09-07

**Authors:** Oguz Kaan Kaya, Zehra Erkal

**Affiliations:** Department of Cardiology, Antalya Training and Research Hospital, University of Health Sciences, 07100 Antalya, Türkiye; zehraerkalkard@hotmail.com

**Keywords:** acute coronary syndrome, angiographic coronary disease severity, Gensini score, uric acid-to-magnesium ratio, frontal QRS-T angle, uric acid-to-HDL cholesterol ratio

## Abstract

**Background:** The uric acid-to-magnesium (UA/Mg) ratio and frontal QRS-T angle have been associated with coronary artery disease, but their relationships with angiographic coronary disease severity in acute coronary syndrome (ACS) remain incompletely characterized. We investigated their associations with the Gensini score in patients with ACS. **Methods:** This single-center, retrospective observational study included 146 patients with ACS undergoing coronary angiography, including 85 with ST-segment elevation myocardial infarction (STEMI) and 61 with non-ST-segment elevation myocardial infarction (NSTEMI). The primary adjusted analysis evaluated the continuous Gensini score after natural logarithmic transformation using multivariable linear regression with HC3 robust standard errors. Median quantile regression was performed as a sensitivity analysis. A Gensini score ≥ 60 was evaluated as a secondary exploratory binary endpoint using a parsimonious multivariable logistic regression model, with bootstrap internal validation. **Results:** The UA/Mg ratio (ρ = 0.332; *p* < 0.001), uric acid-to-HDL cholesterol ratio (UHR) (ρ = 0.232; *p* = 0.005), and frontal QRS-T angle (ρ = 0.481; *p* < 0.001) correlated positively with the Gensini score. In the primary adjusted analysis, both the UA/Mg ratio (β = 0.294 per 1-SD increase; *p* < 0.001) and frontal QRS-T angle (β = 0.225 per 1-SD increase; *p* < 0.001) were independently associated with higher log-transformed Gensini scores. These associations remained significant in median quantile regression and after additional adjustment for C-reactive protein. Modeling uric acid and magnesium separately provided better model fit than use of the UA/Mg ratio. In the secondary exploratory analysis of a Gensini score ≥ 60, the UA/Mg ratio (OR = 1.86 per 1-SD increase; 95% CI: 1.22–2.95; *p* = 0.005) and frontal QRS-T angle (OR = 1.54 per 1-SD increase; 95% CI: 1.05–2.30; *p* = 0.030) remained independently associated with the endpoint. The parsimonious model had an apparent AUC of 0.787 and an optimism-corrected AUC of 0.765 after bootstrap internal validation. **Conclusions:** Higher UA/Mg ratio and wider frontal QRS-T angle were independently associated with a higher log-transformed Gensini score in the primary continuous-outcome analysis in patients with ACS. However, the UA/Mg ratio did not demonstrate statistical superiority over its individual components, and the secondary binary model showed only moderate discrimination after internal validation. These findings should be considered exploratory and require prospective external validation.

## 1. Introduction

Acute coronary syndrome (ACS) encompasses a spectrum of clinical presentations resulting from acute myocardial ischemia, most commonly triggered by atherosclerotic plaque rupture or erosion with superimposed coronary thrombosis. Despite advances in high-sensitivity cardiac troponin testing, antithrombotic therapy, and invasive management, characterization of the anatomical extent of coronary artery disease and overall atherosclerotic burden remains clinically relevant in patients with ACS. The 2023 European Society of Cardiology guidelines address ST-segment elevation myocardial infarction (STEMI) and non-ST-segment elevation acute coronary syndrome within a common disease spectrum and emphasize integrated assessment based on clinical characteristics, cardiac biomarkers, electrocardiographic findings, imaging, and coronary anatomy [1]. Accordingly, readily available biochemical and electrocardiographic markers associated with angiographic coronary disease severity may provide complementary information in the initial evaluation of patients with ACS.

Several angiographic scoring systems have been developed to quantify the anatomical extent and severity of coronary artery disease. Among these, the Gensini score provides a quantitative estimate of angiographic coronary disease severity by integrating the degree of luminal stenosis with the anatomical importance of the affected coronary segment [2]. Higher Gensini scores have been associated with long-term mortality and major adverse cardiovascular events in patients undergoing percutaneous coronary intervention and with an increased risk of periprocedural myocardial infarction [3,4]. Because the Gensini score can only be determined after coronary angiography, there is continued interest in routinely available biomarkers that may be associated with the underlying angiographic disease burden.

Coronary atherosclerosis is a complex process involving inflammation, oxidative stress, endothelial dysfunction, and metabolic dysregulation. In this context, ratio-based biomarkers derived from routine laboratory measurements may integrate complementary biological information. Uric acid, the final product of purine metabolism in humans, is closely related to xanthine oxidoreductase activity. Xanthine oxidoreductase-mediated generation of reactive oxygen species may reduce nitric oxide bioavailability and promote oxidative stress, inflammation, and endothelial dysfunction [5,6]. Elevated serum uric acid levels have also been associated with the presence of coronary artery disease and adverse cardiovascular outcomes [7]. In patients with ACS and hypertension, higher serum uric acid levels have been associated with greater Gensini scores, multivessel coronary disease, and one-year major adverse cardiovascular events [8].

Magnesium is an essential cation involved in energy metabolism, ion-channel function, vascular tone, and maintenance of endothelial integrity. Magnesium deficiency has been linked to endothelial dysfunction and atherosclerotic processes, while data from the long-term Atherosclerosis Risk in Communities (ARIC) study and an accompanying meta-analysis demonstrated an association between lower circulating magnesium concentrations and a higher risk of coronary artery disease [9,10]. Moreover, magnesium status may modify cardiovascular risk in patients with coronary heart disease and hyperuricemia, providing a biological rationale for evaluating uric acid and magnesium jointly [11]. Accordingly, the uric acid-to-magnesium (UA/Mg) ratio may reflect the balance between uric acid-related oxidative and metabolic burden and magnesium-related vascular homeostasis.

In addition to biochemical markers, electrocardiographic indices may provide complementary information regarding myocardial electrical characteristics. The frontal QRS-T angle reflects the difference between ventricular depolarization and repolarization axes and has been associated with myocardial electrical heterogeneity and coronary artery disease severity. UHR represents another readily available metabolic index derived from uric acid and HDL-C. Thus, the UA/Mg ratio, UHR, and frontal QRS-T angle represent metabolic and electrophysiological markers that may be associated with different aspects of coronary disease severity.

In our previous study of patients undergoing elective coronary angiography, the UA/Mg ratio was associated with angiographic coronary disease severity [12]. However, the relationship of the UA/Mg ratio with angiographic disease burden in the acute ischemic setting remains less well-characterized, and ACS introduces additional inflammatory, metabolic, and electrophysiological influences that differ from those encountered in elective angiography populations. The present study was therefore designed as an ACS-specific extension of our previous work in a distinct, non-overlapping cohort rather than as an initial biomarker discovery study. We aimed primarily to evaluate the associations of the UA/Mg ratio and frontal QRS-T angle with angiographic coronary disease severity quantified using the continuous Gensini score in patients with STEMI and NSTEMI. UHR was additionally evaluated as a related uric acid-based index. Secondary exploratory analyses assessed the associations of the UA/Mg ratio and frontal QRS-T angle with a dichotomized Gensini endpoint and the discriminatory performance of a parsimonious model incorporating these markers.

## 2. Materials and Methods

### 2.1. Study Design and Population

**Figure 1:**
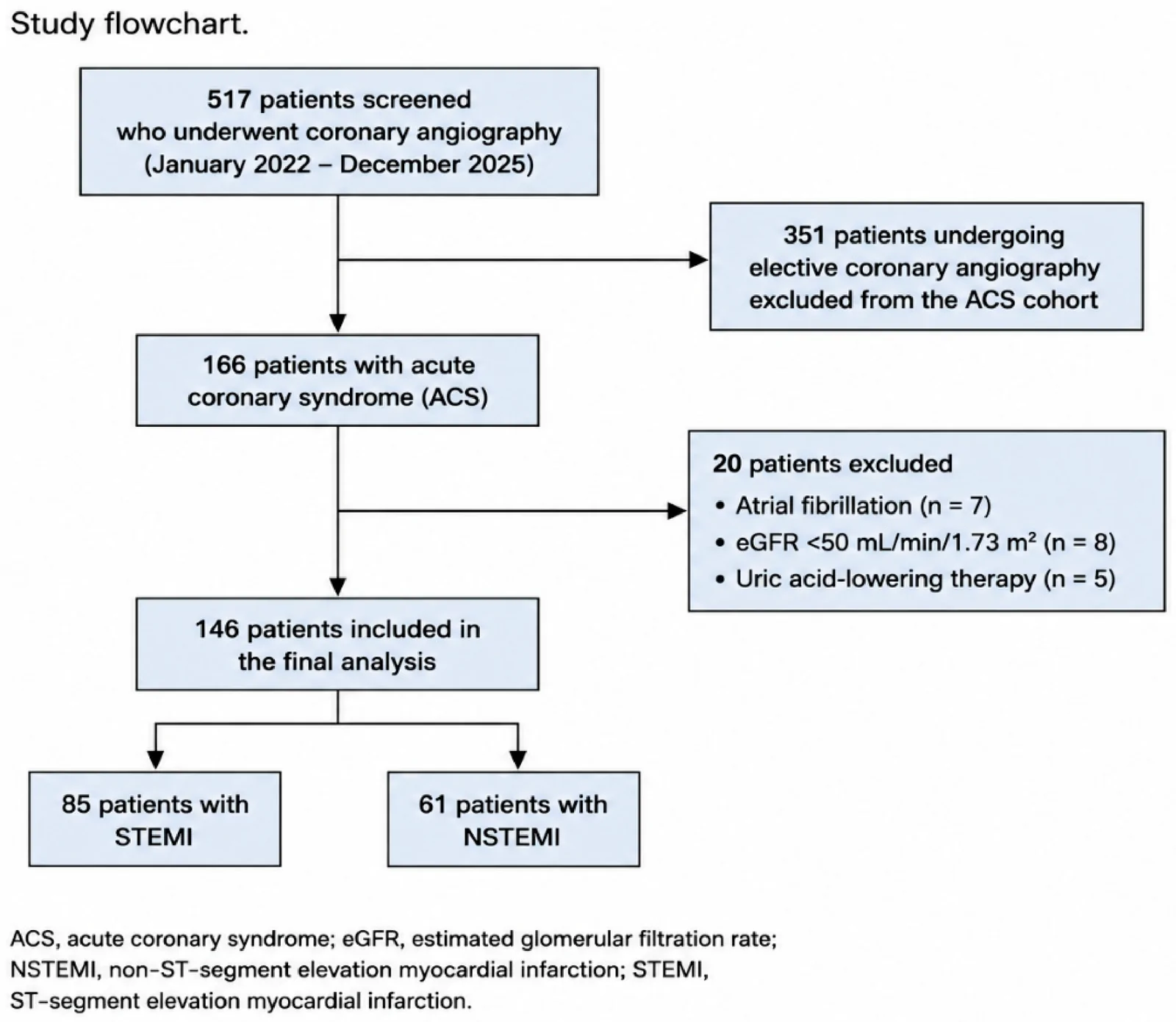
Study flowchart. Of 517 patients undergoing coronary angiography during the study period, 351 undergoing elective coronary angiography were excluded from the ACS cohort. Among 166 patients with ACS, 20 met exclusion criteria, resulting in a final study population of 146 patients (85 STEMI and 61 NSTEMI). ACS, acute coronary syndrome; eGFR, estimated glomerular filtration rate; NSTEMI, non-ST-segment elevation myocardial infarction; STEMI, ST-segment elevation myocardial infarction.

This was a single-center, retrospective observational study conducted at the Cardiology Clinic of the University of Health Sciences Antalya Training and Research Hospital between January 2022 and December 2025. During the study period, 517 consecutive patients undergoing coronary angiography were screened. Of these, 351 patients undergoing elective coronary angiography were excluded because they did not present with acute coronary syndrome (ACS), leaving 166 patients with ACS for eligibility assessment. Patients presenting with ST-segment elevation myocardial infarction (STEMI) or non-ST-segment elevation myocardial infarction (NSTEMI) were retrospectively identified from the hospital’s electronic medical records. Patients were classified according to their ACS presentation as STEMI or NSTEMI. STEMI was defined by the presence of ST-segment elevation on the admission ECG in patients presenting with acute myocardial infarction, whereas patients presenting with myocardial infarction without ST-segment elevation on the admission ECG and with elevated cardiac troponin levels were classified as NSTEMI. After application of the predefined exclusion criteria, 146 patients constituted the final study population. Among the 166 patients with ACS identified during the study period, 20 were excluded from the final analysis: 7 because of atrial fibrillation, 8 because of an eGFR < 50 mL/min/1.73 m^2^, and 5 because of uric acid-lowering therapy. None of the remaining prespecified exclusion criteria resulted in additional exclusions in the screened ACS cohort (Figure 1).

Only the index hospitalization was included for each patient. Patients were excluded if they were younger than 18 years; had previous coronary artery bypass grafting; permanent pacemaker implantation; atrial fibrillation or atrial flutter; complete right or left bundle branch block; severe valvular heart disease; estimated glomerular filtration rate (eGFR) < 50 mL/min/1.73 m^2^; active infection; chronic inflammatory or autoimmune disease; malignancy; severe liver disease; or were receiving uric acid-lowering therapy. Patients with incomplete laboratory, electrocardiographic, or angiographic data required for the study analyses were also excluded.

The study was designed and reported in accordance with the Strengthening the Reporting of Observational Studies in Epidemiology (STROBE) statement. The patient selection process is illustrated in Figure 1.

### 2.2. Clinical and Laboratory Assessment

Demographic characteristics, cardiovascular risk factors, clinical presentation, medical history, and medication use were obtained retrospectively from the hospital’s electronic medical records.

Venous blood samples used for the study analyses were collected as part of routine clinical care at the initial admission to the coronary intensive care unit, before coronary angiography and before the initiation of medical treatment. Serum uric acid, magnesium, creatinine, total cholesterol, low-density lipoprotein cholesterol (LDL-C), high-density lipoprotein cholesterol (HDL-C), triglycerides, and other routine biochemical parameters were measured using standard laboratory methods in the hospital’s central biochemistry laboratory. Estimated glomerular filtration rate (eGFR) was calculated using the Chronic Kidney Disease Epidemiology Collaboration (CKD-EPI) equation.

The uric acid-to-magnesium ratio (UA/Mg) was calculated by dividing serum uric acid by serum magnesium. The uric acid-to-HDL cholesterol ratio (UHR) was calculated by dividing serum uric acid by HDL-C and multiplying the resulting value by 100.

### 2.3. Electrocardiographic Assessment

Standard 12-lead electrocardiograms used for the study analyses were recorded at the time of presentation to the emergency department, before coronary angiography, at a paper speed of 25 mm/s and an amplitude of 10 mm/mV.

The frontal QRS axis and T-wave axis were obtained from automated ECG measurements. The frontal QRS-T angle was calculated as the absolute difference between the frontal QRS and T-wave axes. When the absolute difference exceeded 180°, the frontal QRS-T angle was calculated as 360° minus the absolute difference. A schematic illustration of frontal QRS-T angle determination is provided in Supplementary Figure S1.

All ECGs were independently evaluated by two experienced cardiologists blinded to the angiographic findings. Disagreements were resolved by consensus.

### 2.4. Coronary Angiography and Gensini Score

Coronary angiography was performed by experienced interventional cardiologists using standard radial or femoral approaches. Angiograms were independently reviewed by two interventional cardiologists blinded to the biochemical and electrocardiographic study variables, and disagreements were resolved by consensus. Separate reader-level Gensini scores were not retained in the retrospective dataset; therefore, formal interobserver reproducibility statistics could not be calculated.

Angiographic coronary disease severity was quantified using the Gensini scoring system. Stenosis severity was assigned scores of 1, 2, 4, 8, 16, and 32 for luminal narrowing of 1–25%, 26–50%, 51–75%, 76–90%, 91–99%, and complete occlusion, respectively. These scores were multiplied by weighting factors according to the anatomical importance of the affected coronary segment, and the weighted scores were summed to obtain the total Gensini score.

The primary angiographic outcome was angiographic coronary disease severity quantified using the continuous Gensini score. The total Gensini score included both culprit and non-culprit coronary lesions identified during the index angiography. For secondary exploratory binary analyses, a Gensini score ≥ 60 was used to categorize high angiographic coronary disease severity, consistent with thresholds applied in previous studies [13]. Because no universally accepted or clinically validated Gensini score cutoff defines severe coronary artery disease, the ≥60 threshold was considered an analytical categorization rather than a diagnostic boundary. The robustness of the binary findings to the selected threshold was additionally examined using alternative Gensini score cutoffs of ≥50 and ≥70. In the acute ACS setting, because the total Gensini score included the culprit lesion and could therefore be influenced by acute thrombotic occlusion, it was interpreted as a measure of overall angiographic coronary disease severity.

### 2.5. Statistical Analysis

Statistical analyses were performed using IBM SPSS Statistics for Windows, Version 29.0 (IBM Corp., Armonk, NY, USA) and R Statistical Software, Version 4.5.1 (R Foundation for Statistical Computing, Vienna, Austria).

Normality of continuous variables was assessed using graphical methods and the Shapiro–Wilk test. Normally distributed variables are presented as mean ± standard deviation, non-normally distributed variables as median (interquartile range), and categorical variables as number (percentage). Between-group comparisons were performed using the independent-samples *t*-test or Mann–Whitney U test, as appropriate, whereas categorical variables were compared using the chi-square test or Fisher’s exact test.

Associations of the UA/Mg ratio, UHR, and frontal QRS-T angle with the continuous Gensini score were evaluated using Spearman rank correlation analysis in the overall ACS cohort and separately in the STEMI and NSTEMI subgroups.

Given the non-normal distribution of the Gensini score, the primary adjusted analysis treated the Gensini score as a continuous outcome after natural logarithmic transformation [log(Gensini score + 1)]. Multivariable linear regression with heteroscedasticity-consistent HC3 robust standard errors was used to evaluate the independent associations of the UA/Mg ratio and frontal QRS-T angle with angiographic coronary disease severity. The model included age, sex, diabetes mellitus, hypertension, current smoking, LDL-C, eGFR, left ventricular ejection fraction (LVEF), ACS presentation (STEMI versus NSTEMI), UA/Mg ratio, and frontal QRS-T angle. Continuous predictors were standardized before model fitting. Regression coefficients for the UA/Mg ratio and frontal QRS-T angle are therefore expressed per one-standard-deviation increase. Model explanatory performance was summarized using R^2^ and adjusted R^2^. As a sensitivity analysis that did not require transformation of the outcome, median (τ = 0.50) quantile regression was performed using the same covariates and the untransformed Gensini score.

Because a Gensini score ≥ 60 is not a universally validated clinical threshold, binary analyses were considered secondary and exploratory. To reduce model complexity relative to the number of patients meeting this endpoint, a parsimonious multivariable logistic regression model was constructed including LVEF, ACS presentation (STEMI versus NSTEMI), UA/Mg ratio, and frontal QRS-T angle [14,15]. Continuous predictors were standardized, and odds ratios (ORs) are reported per one-standard-deviation increase with 95% confidence intervals (CIs). The robustness of the findings to the selected Gensini threshold was additionally examined using alternative thresholds of ≥50 and ≥70 with the same parsimonious model.

Receiver operating characteristic (ROC) curve analysis was used to assess the discrimination of the parsimonious model for a Gensini score ≥ 60. Model performance was characterized using the area under the ROC curve (AUC), Brier score, calibration intercept, and calibration slope. Internal validation was performed using 1000 bootstrap resamples to estimate optimism in model performance and to derive optimism-corrected estimates of the AUC, Brier score, and calibration slope.

To assess whether the UA/Mg ratio provided information beyond its individual components, an additional continuous-outcome model was fitted in which serum uric acid and magnesium were entered separately in place of the UA/Mg ratio. Model fit was compared with that of the UA/Mg model using adjusted R^2^ and the Akaike information criterion (AIC). These analyses were intended to assess whether use of the ratio offered statistical advantage over modeling its components separately rather than to establish biological superiority of either approach.

Potential effect modification by ACS presentation was examined in the primary continuous-outcome model by separately introducing interaction terms between ACS subtype (STEMI versus NSTEMI) and the UA/Mg ratio and between ACS subtype and the frontal QRS-T angle. Subgroup-specific correlations were considered descriptive when formal interaction testing did not demonstrate statistically significant effect modification.

Because systemic inflammation may confound the associations of metabolic and electrocardiographic markers with angiographic disease burden, an additional sensitivity analysis was performed by adding log-transformed C-reactive protein (CRP) to the primary continuous-outcome model. Continuous predictors were standardized consistently across multivariable analyses.

A two-sided *p* value < 0.05 was considered statistically significant.

### 2.6. Ethical Approval

This study was conducted in accordance with the Declaration of Helsinki and was approved by the Clinical Research Ethics Committee of the University of Health Sciences Antalya Training and Research Hospital (Approval No. 10/21; Approval Date: 21 May 2026). Owing to the retrospective study design, the requirement for written informed consent was waived by the Ethics Committee.

### 2.7. Use of Generative Artificial Intelligence

Generative artificial intelligence GPT-5.6 Sol (ChatGPT, OpenAI, San Francisco, CA, USA) was used solely to assist with language editing, manuscript organization, and technical drafting of statistical code during manuscript revision. All statistical analyses, numerical outputs, scientific interpretations, and manuscript revisions were independently reviewed and verified by the authors. The authors take full responsibility for the final content of the manuscript.

## 3. Results

### 3.1. Study Population and Baseline Characteristics

A total of 146 consecutive patients with acute coronary syndrome (ACS) who underwent coronary angiography during the study period were included after application of the predefined inclusion and exclusion criteria. Of these, 85 (58.2%) presented with ST-segment elevation myocardial infarction (STEMI) and 61 (41.8%) with non-ST-segment elevation myocardial infarction (NSTEMI). Overall, 46 of 146 patients (31.5%) had a Gensini score ≥ 60. The proportion of patients with a Gensini score ≥ 60 was higher in the STEMI group than in the NSTEMI group (36/85 [42.4%] vs. 10/61 [16.4%]). The patient selection process is illustrated in Figure 1.

The demographic characteristics, cardiovascular risk factors, laboratory findings, electrocardiographic parameters, and angiographic characteristics of the study population are summarized in Table 1. The proportion of male patients was significantly higher in the STEMI group than in the NSTEMI group (84% vs. 66%, *p* = 0.018). No significant between-group differences were observed in age, diabetes mellitus, hypertension, active smoking, serum uric acid, HDL-C, magnesium, LDL-C, creatinine, eGFR, UHR, the UA/Mg ratio, or the frontal QRS-T angle. Left ventricular ejection fraction was significantly lower in the STEMI group (median 50% [45–55] vs. 60% [50–60], *p* < 0.001), whereas the Gensini score was significantly higher (median 48 [32–80] vs. 32 [17–45], *p* < 0.001).

### 3.2. Associations of Biomarkers with the Gensini Score

The associations of the UA/Mg ratio, UHR, and frontal QRS-T angle with the Gensini score were evaluated using Spearman correlation analysis (Table 2). In the overall ACS cohort, the frontal QRS-T angle showed the strongest positive correlation with the Gensini score (ρ = 0.481, *p* < 0.001), followed by the UA/Mg ratio (ρ = 0.332, *p* < 0.001) and UHR (ρ = 0.232, *p* = 0.005).

In the STEMI subgroup, the frontal QRS-T angle (ρ = 0.518, *p* < 0.001) and UA/Mg ratio (ρ = 0.299, *p* = 0.005) were significantly correlated with the Gensini score, while UHR showed a weaker but significant correlation (ρ = 0.232, *p* = 0.032). In the NSTEMI subgroup, the UA/Mg ratio showed the strongest correlation with the Gensini score (ρ = 0.478, *p* < 0.001), while significant correlations were also observed for the frontal QRS-T angle (ρ = 0.364, *p* = 0.004) and UHR (ρ = 0.269, *p* = 0.036). These subgroup-specific correlations were considered descriptive; formal interaction analyses evaluating effect modification by ACS subtype are presented in Section 3.5.

### 3.3. Multivariable Analysis of Continuous Angiographic Coronary Disease Severity

In the primary adjusted analysis, the continuous Gensini score was modeled after natural logarithmic transformation [log(Gensini score + 1)] using multivariable linear regression with HC3 robust standard errors (Table 3). After adjustment for age, sex, diabetes mellitus, hypertension, current smoking, LDL-C, eGFR, LVEF, and ACS subtype, both the UA/Mg ratio (β = 0.294 per 1-SD increase; *p* < 0.001) and frontal QRS-T angle (β = 0.225 per 1-SD increase; *p* < 0.001) were independently associated with a higher log-transformed Gensini score. STEMI presentation was also independently associated with a higher log-transformed Gensini score (β = 0.305; *p* = 0.035). The model explained 37.3% of the variation in log-transformed Gensini score (R^2^ = 0.373; adjusted R^2^ = 0.321).

The findings were consistent in median quantile regression using the untransformed Gensini score. A 1-SD increase in the UA/Mg ratio was associated with a 9.07-point higher median Gensini score (β = 9.07; *p* = 0.002), while a 1-SD increase in the frontal QRS-T angle was associated with a 12.30-point higher median Gensini score (β = 12.30; *p* = 0.013). These results supported the robustness of the associations observed in the primary continuous-outcome analysis.

### 3.4. Secondary Exploratory Analysis of a Gensini Score ≥ 60

Because a Gensini score ≥ 60 is not a universally validated clinical threshold, the binary analysis was considered secondary and exploratory. A parsimonious multivariable logistic regression model including LVEF, ACS presentation, UA/Mg ratio, and frontal QRS-T angle was therefore evaluated. With 46 patients meeting the Gensini ≥ 60 endpoint and four predictor parameters, the model corresponded to approximately 11.5 events per predictor. In this model, STEMI presentation was associated with higher odds of a Gensini score ≥ 60 (OR = 3.73, 95% CI 1.49–10.10; *p* = 0.007), whereas higher LVEF was associated with lower odds (OR = 0.63 per 1-SD increase, 95% CI 0.41–0.96; *p* = 0.036). Both the UA/Mg ratio (OR = 1.86 per 1-SD increase, 95% CI 1.22–2.95; *p* = 0.005) and frontal QRS-T angle (OR = 1.54 per 1-SD increase, 95% CI 1.05–2.30; *p* = 0.030) remained independently associated with the binary endpoint (Table 4).

**Figure 2:**
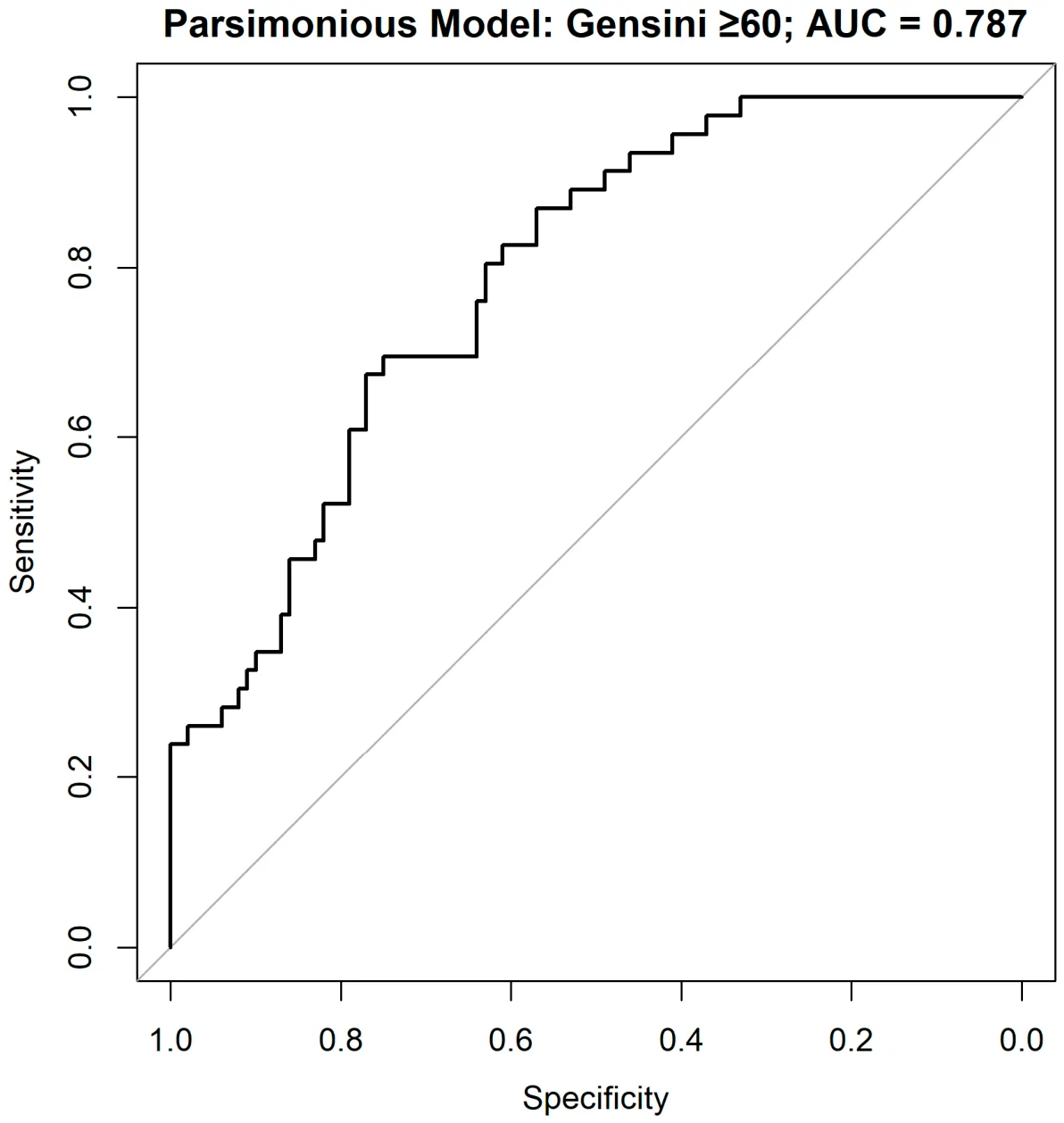
Receiver operating characteristic curve of the parsimonious model for a Gensini score ≥ 60. The model included LVEF, ACS presentation (STEMI versus NSTEMI), UA/Mg ratio, and frontal QRS-T angle. The apparent AUC was 0.787. After internal validation using 1000 bootstrap resamples, the optimism-corrected AUC was 0.765. Because a Gensini score ≥ 60 is not a universally validated clinical threshold, model performance should be interpreted as exploratory. AUC, area under the curve; LVEF, left ventricular ejection fraction; STEMI, ST-segment elevation myocardial infarction; UA/Mg, uric acid-to-magnesium ratio.

The parsimonious model had an apparent AUC of 0.787, an apparent Brier score of 0.169, an apparent calibration intercept of 0.000, and an apparent calibration slope of 1.000 (Figure 2). After 1000 bootstrap resamples, the optimism-corrected AUC was 0.765, the optimism-corrected Brier score was 0.183, and the optimism-corrected calibration slope was 0.875. These findings indicate moderate discrimination with some residual optimism and support interpreting the binary analysis as exploratory rather than as a clinically validated prediction model.

### 3.5. Additional and Sensitivity Analyses

In a sensitivity analysis additionally adjusting the primary continuous-outcome model for log-transformed CRP (Supplementary Table S1), the associations of both the UA/Mg ratio and frontal QRS-T angle with the log-transformed Gensini score remained statistically significant. The UA/Mg ratio remained independently associated with log-transformed Gensini score (β = 0.294 per 1-SD increase; *p* < 0.001), as did the frontal QRS-T angle (β = 0.202 per 1-SD increase; *p* = 0.002). Log-transformed CRP itself was not independently associated with the outcome (β = 0.092; *p* = 0.155). STEMI presentation also remained significant (β = 0.309; *p* = 0.034).

When serum uric acid and magnesium were entered separately instead of the UA/Mg ratio, both uric acid (β = 0.215 per 1-SD increase; *p* < 0.001) and magnesium (β = −0.273 per 1-SD increase; *p* < 0.001) were independently associated with the log-transformed Gensini score. The model containing uric acid and magnesium as separate predictors showed a higher adjusted R^2^ (0.372 vs. 0.321) and a lower AIC (305.68 vs. 316.24) than the model containing the UA/Mg ratio. Thus, the UA/Mg ratio did not demonstrate statistical superiority over modeling its individual components separately.

Formal interaction analyses provided no evidence that the ACS subtype significantly modified the association between the UA/Mg ratio and the log-transformed Gensini score (UA/Mg × STEMI interaction *p* = 0.710) or the association between frontal QRS-T angle and the log-transformed Gensini score (QRS-T × STEMI interaction *p* = 0.106). Therefore, the subgroup-specific correlations observed in STEMI and NSTEMI patients should be interpreted as descriptive rather than as evidence of statistically significant effect modification.

The robustness of the secondary binary findings to the selected Gensini threshold was further examined using alternative cutoffs of ≥50 and ≥70. At a threshold of ≥50 (54 events), both the UA/Mg ratio (OR = 1.71 per 1-SD increase, 95% CI 1.14–2.66; *p* = 0.013) and frontal QRS-T angle (OR = 1.61 per 1-SD increase, 95% CI 1.10–2.43; *p* = 0.018) remained independently associated with the binary endpoint. Similar findings were observed using a threshold of ≥70 (37 events), with corresponding ORs of 1.71 (95% CI 1.12–2.72; *p* = 0.016) for the UA/Mg ratio and 1.67 (95% CI 1.13–2.53; *p* = 0.012) for the frontal QRS-T angle. These findings indicate that the associations observed in the exploratory binary analyses were not dependent on the specific ≥60 threshold.

## 4. Discussion

In this single-center retrospective observational study of patients with ACS, both the UA/Mg ratio and frontal QRS-T angle were independently associated with greater angiographic coronary disease severity when the Gensini score was analyzed as a continuous outcome. These associations remained significant after adjustment for conventional cardiovascular risk factors, renal function, LVEF, and ACS presentation, and were supported by median quantile regression and additional adjustment for CRP. In the primary multivariable analysis, each 1-SD increase in the UA/Mg ratio and frontal QRS-T angle was associated with higher log-transformed Gensini score (β = 0.294 and β = 0.225, respectively; both *p* < 0.001). Importantly, modeling uric acid and magnesium separately provided better model fit than use of the UA/Mg ratio, indicating that the ratio should not be interpreted as statistically superior to its individual components. In the secondary exploratory analysis of a Gensini score ≥ 60, both markers remained independently associated with the binary endpoint in a parsimonious model, which demonstrated moderate discrimination after bootstrap internal validation (optimism-corrected AUC = 0.765). Taken together, these findings support associations of the UA/Mg ratio and frontal QRS-T angle with angiographic disease burden in ACS, while the predictive implications of the binary model remain exploratory and require external validation.

The present findings should be interpreted in the context of our previous study conducted in patients undergoing elective coronary angiography, in which the UA/Mg ratio was associated with angiographic coronary disease severity [12]. The current study was designed as an ACS-specific extension of that work rather than as an initial discovery of the association. Importantly, the present cohort was distinct and non-overlapping and consisted exclusively of patients presenting with STEMI or NSTEMI. This distinction is clinically relevant because the acute ischemic setting introduces additional pathophysiological influences, including acute inflammatory and oxidative responses, myocardial injury, and ischemia-related electrical alterations, that are not equivalently represented in an elective angiography population. The present results therefore provide additional evidence that the association between the UA/Mg ratio and angiographic disease burden is also observed in a distinct, non-overlapping ACS-specific cohort. Nevertheless, the retrospective design and modest sample size warrant interpretation as an extension and replication of previous observations rather than definitive evidence of a novel biomarker.

The association between the UA/Mg ratio and angiographic coronary disease severity is biologically plausible. Uric acid has been linked to xanthine oxidoreductase activity, oxidative stress, reduced nitric oxide bioavailability, endothelial dysfunction, and inflammatory signaling, whereas magnesium contributes to vascular tone, endothelial integrity, ion-channel function, and cellular homeostasis [5,6,9,11]. These distinct biological roles provide a rationale for examining uric acid and magnesium jointly. However, the present analyses do not support statistical superiority of the UA/Mg ratio over its individual components. When uric acid and magnesium were entered separately in the continuous-outcome model, both were independently associated with the log-transformed Gensini score, and the model showed a higher adjusted R^2^ (0.372 vs. 0.321) and a lower AIC (305.68 vs. 316.24) than the model containing the UA/Mg ratio. Thus, although the UA/Mg ratio provides a simple composite measure derived from routinely available laboratory parameters, its association with angiographic disease burden should not be interpreted as evidence that the ratio contains greater statistical information than uric acid and magnesium considered separately.

Previous studies have demonstrated associations between serum uric acid and the presence and severity of coronary artery disease. Zhang et al. reported that elevated serum uric acid levels in patients with ACS and hypertension were associated with higher Gensini scores, multivessel disease, and one-year major adverse cardiovascular events [8]. Similarly, Li et al. reported an association between higher serum uric acid concentrations and the presence of coronary artery disease [7]. These studies evaluated uric acid primarily as an individual biomarker and therefore provide relevant context for the uric acid component of the UA/Mg ratio, but they do not establish whether combining uric acid with magnesium provides additional information beyond the two components considered separately.

Although the magnitude of the correlations differed numerically between the STEMI and NSTEMI subgroups, formal interaction analyses in the primary continuous-outcome model showed no significant effect modification by ACS subtype for either the UA/Mg ratio (UA/Mg × STEMI interaction *p* = 0.710) or the frontal QRS-T angle (QRS-T × STEMI interaction *p* = 0.106). Therefore, the subgroup-specific correlations should be interpreted as descriptive and hypothesis-generating rather than as evidence that the associations differ between STEMI and NSTEMI patients.

UHR represents another uric acid-based composite index that combines uric acid with HDL-C. Yaman et al. reported significant associations of UHR with both SYNTAX and Gensini scores [16]. In the present study, UHR was positively correlated with the continuous Gensini score, supporting an association with angiographic coronary disease severity. However, because the revised multivariable analyses were focused on the UA/Mg ratio and frontal QRS-T angle as the prespecified markers of primary interest, the present findings do not permit conclusions regarding the independent or incremental association of UHR with angiographic coronary disease severity relative to these markers.

Systemic inflammation represents an important potential confounder in interpreting these associations. Inflammation plays a central role in atherosclerotic plaque progression, plaque instability, and ACS and may influence both metabolic biomarkers and angiographic disease burden. Recent evidence has demonstrated distinct inflammatory phenotypes among patients undergoing percutaneous coronary intervention, with substantial differences in subsequent cardiovascular risk [17]. In the sensitivity analysis additionally adjusting the primary continuous-outcome model for log-transformed CRP, both the UA/Mg ratio (β = 0.294 per 1-SD increase; *p* < 0.001) and frontal QRS-T angle (β = 0.202 per 1-SD increase; *p* = 0.002) remained independently associated with the log-transformed Gensini score, whereas log-transformed CRP itself was not independently associated with the outcome (β = 0.092; *p* = 0.155). These findings suggest that the observed associations were not materially attenuated by adjustment for CRP. Nevertheless, CRP captures only one component of the inflammatory response, and residual confounding by systemic inflammation cannot be excluded.

The frontal QRS-T angle was also independently associated with a higher log-transformed Gensini score in the primary continuous-outcome analysis. The frontal QRS-T angle reflects the spatial discordance between ventricular depolarization and repolarization axes and has been associated with myocardial electrical heterogeneity, ischemia, fibrosis, ventricular remodeling, and adverse cardiovascular outcomes [18,19,20,21,22,23]. In a meta-analysis including 22 studies and 164,171 participants, Zhang et al. demonstrated that wider frontal or spatial QRS-T angles were associated with all-cause and cardiac mortality [18]. Akın and Bilge reported that the frontal QRS-T angle was higher in patients with greater angiographic coronary disease severity as assessed by the Gensini score [19], while Karadeniz and Altuntaş demonstrated an association between the frontal QRS-T angle and higher SYNTAX scores in stable coronary artery disease [20].

Evidence from ACS populations also supports the clinical relevance of the frontal QRS-T angle. Lown et al. reported that a frontal QRS-T angle–age risk score derived from the admission ECG was associated with mortality in patients with ACS [21]. Küçük demonstrated an association between the frontal QRS-T angle, impaired baseline coronary flow, and in-hospital adverse events in NSTEMI [22]. More recently, Özkoç et al. reported that the frontal QRS-T angle was independently associated with the no-reflow phenomenon in patients with ACS [23].

However, interpretation of the frontal QRS-T angle in the present study requires particular caution because ECGs were obtained at the time of presentation to the emergency department, before coronary angiography, during the acute ACS presentation. Acute myocardial ischemia, infarct location, ischemia-related ST-T changes, the magnitude of myocardial injury, and the timing of ECG acquisition relative to symptom onset may independently influence ventricular repolarization and thereby widen the frontal QRS-T angle. In parallel, the total Gensini score obtained during the index angiography includes the culprit lesion, and severe or completely occluded culprit lesions, particularly in STEMI, may substantially contribute to the total angiographic score. Consequently, the observed association between the frontal QRS-T angle and Gensini score may partly reflect the severity and electrophysiological consequences of the acute ischemic event rather than the extent of pre-existing coronary disease alone. Although the association remained significant after adjustment for ACS subtype, and formal interaction testing did not demonstrate significant effect modification by STEMI versus NSTEMI presentation, these analyses cannot fully eliminate confounding related to acute infarct characteristics. Culprit-lesion-excluded Gensini scores could not be reliably reconstructed from the retrospective data because standardized lesion-level information required for such recalculation was not available. Future prospective studies incorporating culprit and non-culprit disease burden, infarct territory, pre-intervention coronary flow, peak cardiac troponin, and symptom-to-ECG time are needed to clarify the independent relationship between the frontal QRS-T angle and pre-existing coronary disease severity.

The UA/Mg ratio and frontal QRS-T angle represent different biological domains, but the discriminatory performance of a model incorporating both markers should be interpreted cautiously. In the secondary exploratory analysis of a Gensini score ≥ 60, a parsimonious model incorporating LVEF, ACS presentation, UA/Mg ratio, and frontal QRS-T angle achieved an apparent AUC of 0.787. In the development dataset, the apparent calibration intercept and slope were 0.000 and 1.000, respectively. After 1000 bootstrap resamples, the optimism-corrected AUC was 0.765, with an optimism-corrected Brier score of 0.183 and an optimism-corrected calibration slope of 0.875. These findings indicate moderate discrimination with some residual optimism rather than evidence of a clinically validated prediction model. Moreover, because a Gensini score ≥ 60 is not a universally validated clinical threshold and the study lacked an independent external validation cohort, the model should be regarded as exploratory and hypothesis-generating. Its potential clinical utility cannot be established from the present data.

Previous multimarker studies provide relevant context for these findings. Biomarkers representing different pathobiological pathways may contribute to cardiovascular risk assessment after acute myocardial infarction and ACS [24,25]. However, improvements in statistical model fit do not necessarily translate into clinically meaningful gains in risk prediction, and many candidate biomarkers lose independent value after comprehensive clinical adjustment [26,27]. These observations reinforce the need to distinguish statistical association and model discrimination from demonstrated clinical utility.

The potential clinical role of the UA/Mg ratio and frontal QRS-T angle should be considered within the context of contemporary ACS management. Patients with STEMI and high-risk NSTEMI generally undergo invasive coronary evaluation according to established clinical indications; therefore, these markers should not be viewed as tools for determining whether coronary angiography is required. Rather, their observed associations may provide information regarding angiographic coronary disease severity. However, given the retrospective design, modest sample size, use of a non-universally validated Gensini ≥60 threshold for the secondary binary analysis, and absence of external validation, the present findings do not establish clinical utility or support changes in patient management. Whether these readily available markers provide clinically meaningful information beyond established clinical risk assessment requires prospective evaluation and external validation.

Several strengths of the present study should be acknowledged. The study evaluated metabolic and electrocardiographic markers in the same ACS cohort in relation to angiographic coronary disease severity quantified using the Gensini score. Importantly, the primary adjusted analysis treated the Gensini score as a continuous outcome rather than relying on an arbitrary dichotomization and used HC3 robust standard errors, with consistent findings obtained in median quantile regression. Additional analyses included adjustment for CRP, formal interaction testing by ACS subtype, and direct comparison of the UA/Mg ratio with uric acid and magnesium modeled separately. The latter analysis demonstrated that the ratio was not statistically superior to its individual components, providing a more balanced interpretation of its potential value. For the secondary exploratory Gensini ≥60 endpoint, model complexity was restricted to four predictor parameters, corresponding to approximately 11.5 events per predictor, and bootstrap internal validation was used to quantify model optimism. Nevertheless, the optimism-corrected AUC of 0.765 indicates only moderate discrimination, and no independent external validation cohort was available. Therefore, the reproducibility and potential clinical relevance of these findings require confirmation in larger prospective multicenter studies.

## 5. Limitations

This study has several limitations. First, its retrospective, single-center design and relatively modest sample size limit causal inference and generalizability. No prospective sample-size calculation was performed because the study included eligible patients identified retrospectively during the predefined study period. Although the primary analysis treated the Gensini score as a continuous outcome, the secondary binary analysis was based on a Gensini score ≥ 60, which is not a universally validated clinical threshold and should therefore be considered exploratory. To reduce overfitting in this secondary analysis, the logistic model was restricted to four predictor parameters for 46 events, and bootstrap internal validation was performed; nevertheless, the optimism-corrected AUC of 0.765 and calibration slope of 0.875 indicate moderate discrimination and some residual model optimism, and no independent external validation cohort was available.

Second, angiographic coronary disease severity was quantified using the Gensini score, which is an angiographic surrogate and does not provide the lesion-level morphological or physiological information obtainable from intravascular imaging or functional assessment. In addition, the total Gensini score included the culprit lesion. Particularly in STEMI, severe stenosis or complete occlusion of the culprit vessel may substantially contribute to the total score; therefore, the measured Gensini score may reflect both pre-existing coronary disease and characteristics of the acute ischemic event. Culprit-lesion-excluded Gensini scores could not be reliably reconstructed because standardized lesion-level information required for retrospective recalculation was unavailable. Although coronary angiograms were independently evaluated by two interventional cardiologists and disagreements were resolved by consensus, separate reader-level Gensini scores were not retained in the retrospective dataset. Therefore, formal interobserver reproducibility statistics, such as the intraclass correlation coefficient, could not be calculated retrospectively.

Third, the frontal QRS-T angle was derived from ECGs obtained during the acute ACS presentation and may have been influenced by acute myocardial ischemia, infarct location, ischemia-related ST-T changes, the magnitude of myocardial injury, and the timing of ECG acquisition relative to symptom onset. Detailed information on infarct territory, pre-intervention coronary flow, peak cardiac troponin, and symptom-to-ECG time was not sufficiently standardized in the retrospective dataset to permit comprehensive adjustment. Consequently, residual confounding related to acute infarct severity cannot be excluded, and the association between the frontal QRS-T angle and Gensini score should not be interpreted as reflecting pre-existing coronary disease severity alone.

Fourth, biomarkers were assessed at hospital admission, and serial changes during hospitalization were not evaluated. Although the associations of both the UA/Mg ratio and frontal QRS-T angle remained significant after additional adjustment for CRP in the continuous-outcome model, CRP captures only one component of the inflammatory response, and residual confounding by systemic inflammation and other unmeasured factors remains possible. Information on dietary magnesium intake, magnesium supplementation, and other lifestyle-related determinants was also unavailable. Moreover, modeling uric acid and magnesium separately provided better statistical fit than use of the UA/Mg ratio; therefore, the present study does not establish statistical superiority of the ratio over its individual components.

Fifth, the exclusion of patients with conditions that could substantially affect biochemical or electrocardiographic measurements, including atrial fibrillation, bundle branch block, pacemaker rhythm, reduced renal function, inflammatory disease, malignancy, and urate-lowering therapy, resulted in a selected ACS population. Although these exclusions reduced important sources of measurement distortion and confounding, they may limit generalizability, particularly to patients with chronic kidney disease, atrial fibrillation, or conduction abnormalities. The findings also may not be generalizable to patients with unstable angina, chronic coronary syndromes, or ACS managed without an invasive strategy.

Finally, the study evaluated angiographic coronary disease severity rather than longitudinal clinical outcomes. Accordingly, the present findings do not establish the prognostic value of the UA/Mg ratio or frontal QRS-T angle for major adverse cardiovascular events or mortality and do not support their use for clinical decision-making. Larger prospective multicenter studies incorporating standardized assessment of acute infarct characteristics, culprit and non-culprit coronary disease, serial biomarker measurements, longitudinal clinical outcomes, and independent external validation are required to confirm these findings and determine their potential clinical relevance.

## 6. Conclusions

In patients with acute coronary syndrome, higher UA/Mg ratio and wider frontal QRS-T angle were independently associated with a higher log-transformed Gensini score in the primary continuous-outcome analysis. These associations remained robust in sensitivity analyses, including median quantile regression and additional adjustment for CRP. However, the UA/Mg ratio did not demonstrate statistical superiority over uric acid and magnesium modeled separately. In the secondary exploratory analysis of a Gensini score ≥ 60, both markers remained independently associated with the binary endpoint within a parsimonious model that showed moderate discrimination after bootstrap internal validation. Given the retrospective single-center design, modest sample size, non-universally validated binary threshold, and absence of external validation, these findings should be considered exploratory and hypothesis-generating. Prospective multicenter studies are required to confirm these associations and determine whether they provide clinically meaningful information beyond established risk assessment.

## Figures and Tables

**Table 1:** Baseline characteristics of patients according to acute coronary syndrome subtype (STEMI vs. NSTEMI).

Variable	Overall (n = 146)	STEMI (n = 85)	NSTEMI (n = 61)	*p* Value
Age, years	57 (51–69)	57 (51–68)	58 (51–70)	0.340
Male sex, n (%)	111 (76%)	71 (84%)	40 (66%)	0.018
Diabetes mellitus, n (%)				0.866
No	82 (56%)	47 (55%)	35 (57%)	
Yes	64 (44%)	38 (45%)	26 (43%)	
Hypertension, n (%)				0.310
No	63 (43%)	40 (47%)	23 (38%)	
Yes	83 (57%)	45 (53%)	38 (62%)	
Current smoking, n (%)				0.733
No	55 (38%)	31 (36%)	24 (39%)	
Yes	91 (62%)	54 (64%)	37 (61%)	
LVEF, %	50.0 (45.0–60.0)	50.0 (45.0–55.0)	60.0 (50.0–60.0)	<0.001
Uric acid, mg/dL	5.5 (4.6–6.8)	5.3 (4.5–6.5)	5.9 (4.9–7.2)	0.070
HDL cholesterol, mg/dL	43.0 (36.0–49.0)	42.0 (37.0–48.0)	44.0 (36.0–51.0)	0.503
Magnesium, mg/dL	1.90 (1.80–2.10)	1.90 (1.70–2.00)	1.90 (1.80–2.10)	0.122
LDL cholesterol, mg/dL	130.5 (108.0–154.0)	131.0 (110.0–154.0)	128.0 (99.0–154.0)	0.637
Creatinine, mg/dL	0.95 (0.85–1.06)	0.95 (0.85–1.03)	0.93 (0.82–1.11)	0.983
eGFR, mL/min/1.73 m^2^	83.0 (74.0–91.0)	84.0 (76.0–90.0)	82.0 (68.0–92.0)	0.465
Uric acid-to-HDL ratio	12.92 (10.58–16.76)	12.67 (10.75–16.17)	13.19 (10.55–17.14)	0.522
Uric acid-to-magnesium ratio	2.97 (2.56–3.60)	2.86 (2.56–3.52)	3.14 (2.55–3.76)	0.281
Frontal QRS-T angle, degrees	44.0 (31.0–70.0)	44.0 (33.0–74.0)	42.0 (29.0–60.0)	0.255
Gensini score	39.5 (24.0–71.0)	48.0 (32.0–80.0)	32.0 (17.0–45.0)	<0.001

Footnote. Continuous variables are presented as median (interquartile range) and categorical variables as number (percentage). *p* values were calculated using the Mann–Whitney U test for continuous variables and the chi-square test or Fisher’s exact test, as appropriate, for categorical variables. eGFR, estimated glomerular filtration rate; HDL, high-density lipoprotein; LDL, low-density lipoprotein; LVEF, left ventricular ejection fraction; NSTEMI, non-ST-segment elevation myocardial infarction; STEMI, ST-segment elevation myocardial infarction.

**Table 2:** Spearman correlation analysis between biomarkers and Gensini score in the overall acute coronary syndrome cohort and predefined subgroups.

Variable	Overall, ρ (*p* Value)	STEMI, ρ (*p* Value)	NSTEMI, ρ (*p* Value)
Uric acid-to-HDL ratio (UHR)	0.232 (0.005)	0.232 (0.032)	0.269 (0.036)
Uric acid-to-magnesium ratio (UA/Mg)	0.332 (<0.001)	0.299 (0.005)	0.478 (<0.001)
Frontal QRS-T angle	0.481 (<0.001)	0.518 (<0.001)	0.364 (0.004)

Footnote. Correlation coefficients (ρ) were calculated using Spearman’s rank correlation analysis. A two-sided *p* value < 0.05 was considered statistically significant. NSTEMI, non-ST-segment elevation myocardial infarction; STEMI, ST-segment elevation myocardial infarction; UA/Mg, uric acid-to-magnesium ratio; UHR, uric acid-to-high-density lipoprotein cholesterol ratio.

**Table 3:** Multivariable linear regression analysis of factors associated with continuous angiographic coronary disease severity.

Variable	β Coefficient	Robust SE (HC3)	*p* Value
Age (per 1 SD increase)	−0.029	0.069	0.680
Male sex	0.028	0.135	0.834
Diabetes mellitus	−0.047	0.143	0.742
Hypertension	−0.182	0.125	0.149
Current smoking	−0.202	0.147	0.173
LDL cholesterol (per 1 SD increase)	−0.022	0.056	0.692
eGFR (per 1 SD increase)	−0.027	0.075	0.721
LVEF (per 1 SD increase)	−0.123	0.068	0.070
STEMI	0.305	0.143	0.035
UA/Mg ratio (per 1 SD increase)	0.294	0.067	<0.001
Frontal QRS-T angle (per 1 SD increase)	0.225	0.062	<0.001

Footnote. The dependent variable was the natural logarithm of the Gensini score plus 1 [log(Gensini score + 1)]. Multivariable linear regression was performed using heteroscedasticity-consistent HC3 robust standard errors. Continuous predictors were standardized, and β coefficients for these variables are expressed per one-standard-deviation increase. R^2^ = 0.373; adjusted R^2^ = 0.321. eGFR, estimated glomerular filtration rate; LDL, low-density lipoprotein; LVEF, left ventricular ejection fraction; STEMI, ST-segment elevation myocardial infarction; UA/Mg, uric acid-to-magnesium ratio.

**Table 4:** Parsimonious multivariable logistic regression analysis for a Gensini score ≥ 60.

Variable	OR	95% CI	*p* Value
LVEF (per 1-SD increase)	0.63	0.41–0.96	0.036
STEMI	3.73	1.49–10.10	0.007
UA/Mg ratio (per 1-SD increase)	1.86	1.22–2.95	0.005
Frontal QRS-T angle (per 1-SD increase)	1.54	1.05–2.30	0.030

Footnote. The binary endpoint was a Gensini score ≥ 60 and was considered secondary and exploratory because this threshold is not a universally validated clinical cutoff. Continuous predictors were standardized before model fitting; ORs for LVEF, UA/Mg ratio, and frontal QRS-T angle are therefore expressed per one-standard-deviation increase. The model included 46 events and four predictor parameters (approximately 11.5 events per predictor). OR, odds ratio; CI, confidence interval; LVEF, left ventricular ejection fraction; STEMI, ST-segment elevation myocardial infarction; UA/Mg, uric acid-to-magnesium ratio.

## Data Availability

The data supporting the findings of this study are available within the article. Additional anonymized data are available from the corresponding author upon reasonable request, subject to institutional and ethical regulations.

## Supplementary Materials

The following supporting information can be downloaded at: https://www.mdpi.com/article/10.3390/jcm15176920/s1, Table S1. Additional and sensitivity analyses of angiographic coronary atherosclerotic burden; Figure S1. Schematic illustration of frontal QRS-T angle determination.
