# Supplementary material for: Association of the Uric Acid-to-Magnesium Ratio and Frontal QRS-T Angle with Angiographic Coronary Disease Severity in Acute Coronary Syndrome
Source: J Clin Med. 2026 Sep 7;15(17):6920. doi: 10.3390/jcm15176920 (PMC13566105; doi:10.3390/jcm15176920)
Supplement: Supplementary file 1 [file jcm-15-06920-s001.zip › jcm-4553930-supplementary.pdf]

Supplementary Table S1. Additional and sensitivity analyses of angiographic coronary atherosclerotic burden.

A. **Comparison of the UA/Mg ratio with its individual components**

Model	Adjusted R^2	AIC
Primary model including UA/Mg ratio	0.321	316.24
Model including uric acid and magnesium separately	0.372	305.68

In the separate-component model, uric acid (β = 0.215 per 1-SD increase; $p < 0.001$) and magnesium (β = −0.273 per 1-SD increase; $p < 0.001$) were independently associated with the log-transformed Gensini score.

B. **Interaction analyses according to ACS subtype**

Interaction term	β coefficient	p value
ACS subtype × UA/Mg ratio	0.051	0.710
ACS subtype × frontal QRS-T angle	0.228	0.106

C. **Sensitivity analysis additionally adjusted for CRP**

Variable	β coefficient	p value
UA/Mg ratio, per 1-SD increase	0.294	<0.001
Frontal QRS-T angle, per 1-SD increase	0.202	0.002
STEMI	0.309	0.034
Log-transformed CRP	0.092	0.155

D. Alternative Gensini thresholds

Threshold	Events	UA/Mg ratio OR (95% CI)	p value	Frontal QRS-T angle OR (95% CI)	p value
Gensini ≥50	54	1.71 (1.14–2.66)	0.013	1.61 (1.10–2.43)	0.018

Threshold	Events	UA/Mg ratio OR (95% CI)	p value	Frontal QRS-T angle OR (95% CI)	p value
Gensini ≥70	37	1.71 (1.12–2.72)	0.016	1.67 (1.13–2.53)	0.012

E. Bootstrap internal validation of the parsimonious Gensini ≥60 model

Performance measure	Apparent value	Optimism-corrected value
AUC	0.787	0.765
Brier score	0.169	0.183
Calibration slope	1.000	0.875

Footnote: The primary continuous-outcome model used log-transformed Gensini score [log(Gensini score + 1)] and included age, sex, diabetes mellitus, hypertension, current smoking, LDL cholesterol, eGFR, LVEF, ACS subtype (STEMI versus NSTEMI), UA/Mg ratio, and frontal QRS-T angle. Continuous predictors were standardized before model fitting. The CRP sensitivity analysis additionally included log-transformed CRP. Alternative-threshold analyses used the parsimonious logistic regression model including LVEF, ACS subtype, UA/Mg ratio, and frontal QRS-T angle; odds ratios for continuous predictors are expressed per one-standard-deviation increase. Bootstrap internal validation of the Gensini ≥60 model was based on 1000 resamples. AIC, Akaike information criterion; ACS, acute coronary syndrome; AUC, area under the receiver operating characteristic curve; CI, confidence interval; CRP, C-reactive protein; eGFR, estimated glomerular filtration rate; LDL, low-density lipoprotein; LVEF, left ventricular ejection fraction; OR, odds ratio; STEMI, ST-segment elevation myocardial infarction; UA/Mg, uric acid-to-magnesium ratio.

Supplementary Figure S1

Supplementary Figure S1. Schematic illustration of frontal QRS-T angle determination.

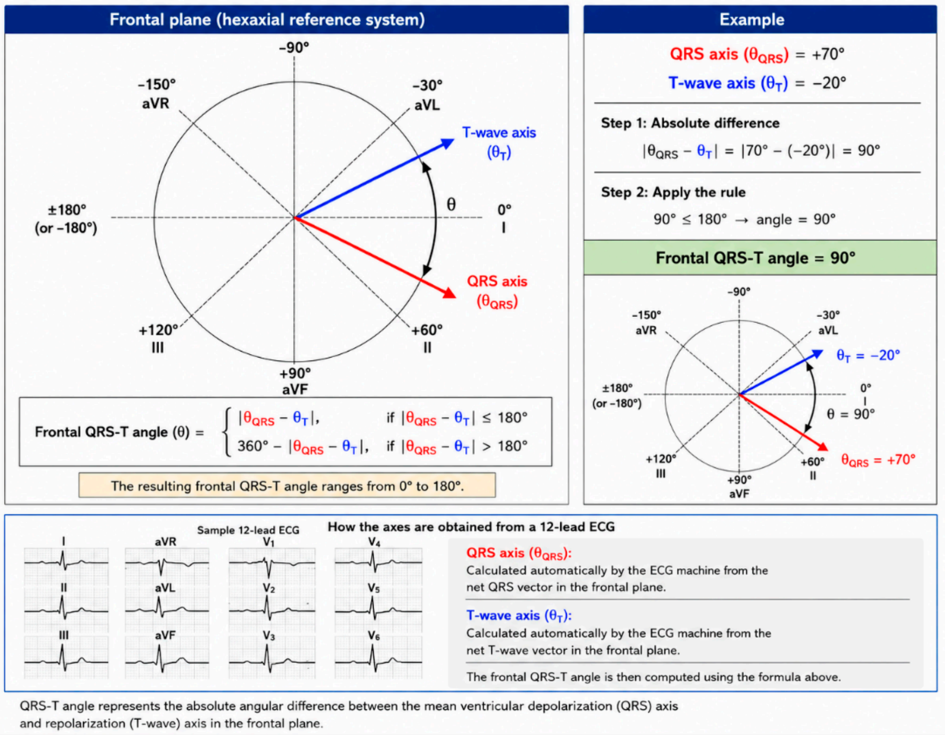

Supplementary Figure S1. Schematic illustration of frontal QRS-T angle determination. The frontal QRS-T angle was calculated as the absolute difference between the frontal QRS axis and T-wave axis obtained from the standard 12-lead electrocardiogram. When the absolute difference exceeded 180°, the frontal QRS-T angle was calculated as 360° minus the absolute difference, yielding an angle ranging from 0° to 180°. QRS, ventricular depolarization; T, ventricular repolarization.
